# Dose escalation study to evaluate safety, tolerability and efficacy of intravenous etoposide phosphate administration in 27 dogs with multicentric lymphoma

**DOI:** 10.1371/journal.pone.0177486

**Published:** 2017-05-15

**Authors:** Pierre Boyé, François Serres, Laurent Marescaux, Juliette Hordeaux, Emmanuel Bouchaert, Bruno Gomes, Dominique Tierny

**Affiliations:** 1 Oncovet-Clinical-Research (OCR), SIRIC ONCOLille, Parc Eurasanté, Loos, France; 2 Oncovet, SIRIC ONCOLille, Villeneuve d’Ascq, France; 3 Institut de Recherche Pierre Fabre, Toulouse, France; Colorado State University, UNITED STATES

## Abstract

Comparative oncology has shown that naturally occurring canine cancers are of valuable and translatable interest for the understanding of human cancer biology and the characterization of new therapies. This work was part of a comparative oncology project assessing a new, clinical-stage topoisomerase II inhibitor and comparing it with etoposide in dogs with spontaneous lymphoma with the objective to translate findings from dogs to humans. Etoposide is a topoisomerase II inhibitor widely used in various humans’ solid and hematopoietic cancer, but little data is available concerning its potential antitumor efficacy in dogs. Etoposide phosphate is a water-soluble prodrug of etoposide which is expected to be better tolerated in dogs. The objectives of this study were to assess the safety, the tolerability and the efficacy of intravenous etoposide phosphate in dogs with multicentric lymphoma. Seven dose levels were evaluated in a traditional 3+3 phase I design. Twenty-seven owned-dogs with high-grade multicentric lymphoma were enrolled and treated with three cycles of etoposide phosphate IV injections every 2 weeks. Adverse effects were graded according to the Veterinary Cooperative Oncology Group criteria. A complete end-staging was realized 45 days after inclusion. The maximal tolerated dose was 300 mg/m^2^. At this dose level, the overall response rate was 83.3% (n = 6, 3 PR and 2 CR). Only a moderate reversible gastrointestinal toxicity, no severe myelotoxicity and no hypersensitivity reaction were reported at this dose level. Beyond the characterization of etoposide clinical efficacy in dogs, this study underlined the clinical and therapeutic homologies between dog and human lymphomas.

## Introduction

Non-Hodgkin lymphoma is the seventh most common human systemic malignancy, with an estimated prevalence of 70,000 patients in the United States in 2013 [1]. The addition of the anti-CD20 monoclonal antibody therapy to cyclophosphamide, doxorubicin, vincristine, and prednisone (CHOP) greatly improved the prognosis of diffuse large B-cell lymphoma (DLBCL), but nearly one-third of patients do not achieve durable remission and develop relapsed/refractory disease, underlining the considerable possibility for therapeutic improvement [2].

Etoposide, a semisynthetic derivative of podophyllotoxine, is a cytotoxic chemotherapy drug mediated by inhibition of topoisomerase II [3]. Etoposide has been used for many years in humans for refractory or relapsed non-Hodgkin lymphoma in combination with other drugs [3–5].

This dose escalation study was determined as part of a comparative oncology project to compare the safety and the efficacy of F14512, a new polyamine-vectorized, podophyllotoxine-derivative anticancer drug, and etoposide phosphate in dogs with naturally occurring high-grade lymphoma. F14512 is a vectorized form of etoposide and has demonstrated *in vitro* and *in vivo* potent, superior to etoposide, antitumor activities in preclinical studies [6–13]. Comparative oncology has revealed that spontaneous lymphomas in dogs share clinical, biologic, genetic, and therapeutic similarities with their human counterparts [14,15]. A phase I clinical pharmacology study of F14512 in naturally occurring canine lymphoma was previously published to determine the recommended dose of F14512 in dogs [16]. For comparative purposes, the safety, the tolerability and the efficacy of intravenous etoposide phosphate needed to be investigated in order to determine the recommended dose in dogs with multicentric lymphoma.

Etoposide is widely used in various humans’ solid and hematopoietic cancers [3,17,18], but little data is available concerning its potential antitumor efficacy in dogs [19,20]. Etoposide has been studied in dogs for pharmacokinetics and toxicologic purposes [21,22]. Previous studies on canine lymphoma treated with intravenous administration of etoposide showed a minimal therapeutic effect associated with hematologic toxicity and severe acute hypersensitivity reactions probably associated with the vehicle (polysorbate-80) used for the parenteral formulation [23]. This vehicle-related toxicosis was considered a limitation for the IV administration of etoposide in dogs. In humans, intravenous etoposide is generally well tolerated, but rare acute hypersensitivity reactions have been reported, manifested by dyspnea, chest discomfort, hypotension, bronchospasm and skin flushing [24,25]. Patients who initially experienced a hypersensitivity reaction to intravenous etoposide tolerated the subsequent administration of intravenous etoposide phosphate without any allergic reaction [24,25].

Etoposide phosphate is a water soluble phosphate ester derivative of etoposide which does not contain polysorbate-80. This IV formulation is expected to be easier to use and better tolerated in dogs. *In vivo*, etoposide phosphate is rapidly and extensively converted to etoposide by phosphatases present in serum and tissues [21]. In murine models, this pro-drug has shown a similar activity to etoposide in various tumors [26].

The objectives of this dose escalation clinical trial were (i) to assess the clinical and hematologic tolerance and determine the recommended dose of intravenous etoposide phosphate (ETOPOPHOS^®^) in dogs with naturally occurring high-grade lymphoma; and (ii) to identify early signs of efficacy of this treatment, with an evaluation of remission rate after three cycles of therapy.

This dose escalation study is a pilot translational research study for a future phase II randomized double-blind clinical study of F14512 and etoposide phosphate in naturally occurring canine lymphoma.

## Materials and methods

The study design was prospective, open-labelled and monocentric. The study was conducted by Oncovet Clinical Research (OCR) as part of a collaborative research project between OCR and Pierre Fabre Medicament.

### Dose escalation design

The aim of the dose escalation study was to assess the safety and tolerability of etoposide phosphate in order to determine the recommended dose in dogs. A traditional 3+3 phase I design was used for the dose escalation study [27]. In each cohort, a minimum of 3 dogs were included and received the same dose during the protocol. The first cohort was treated at a starting dose that was considered to be safe based on extrapolation from toxicological data [21] and the next cohorts were treated at increasing dose levels according to the following criteria: if no Dose Limiting Toxicity (DLT) was observed in the 3 dogs treated in a cohort, another three dogs could be treated at the next higher dose level. If one of the three dogs experienced a DLT, three more dogs were treated at the same dose level. The dose escalation was continued until at least two dogs among a cohort of three or six dogs experienced a DLT. The recommended dose was defined as the dose level just below this toxic dose level.

In addition, after the last dog was included in the dose escalation study, three more dogs were included and treated with the maximal tolerated dose to assess early signs of efficacy at this dose level.

### Treatment schedule

Seven dose levels were evaluated (Table 1): 105 mg/m^2^ (cohort 1; n = 3), 126 mg/m^2^ (cohort 2; n = 5), 150 mg/m^2^ (cohort 3; n = 4), 180 mg/m^2^ (cohort 4; n = 3), 225 mg/m^2^ (cohort 5; n = 4), 300 mg/m^2^ (cohort 6; n = 6) and 360 mg/m^2^ (cohort 7; n = 2). All dogs involved in the study followed the same protocol over a period of 6 weeks.

**Table 1:** Dose escalation schedule. The first cohort was treated at a starting dose of 105 mg/m^2^ (daily dose of 35 mg/m^2^) that was considered to be safe based on extrapolation from toxicological data in dogs [21]. In the three following dose levels, a 20%- increase dose level was used. Due to the good tolerance at the dose levels n°1, 2, 3 and 4 but a minimal therapeutic effect observed, it was decided to treat dogs with a 25% and then a 33%-increase dose level in cohort n°5 and 6 respectively in order to reach the daily dose of 100 mg/m^2^ reported in humans [28]. In the cohort n°7, dogs were treated with the initial 20%-increase dose level.

Cohort No	Single Dose	Total dose / cycle	% Dose escalation
1	35 mg/m^2^	105 mg/m^2^	Baseline
2	42 mg/m^2^	126 mg/m^2^	20%
3	50 mg/m^2^	150 mg/m^2^	19%
4	60 mg/m^2^	180 mg/m^2^	20%
5	75 mg/m^2^	225 mg/m^2^	25%
6	100 mg/m^2^	300 mg/m^2^	33%
7	120 mg/m^2^	360 mg/m^2^	20%

For comparison purposes with the final phase II randomized double-blind clinical study of F14512 and etoposide phosphate, the recommended dose of etoposide phosphate was determined with the same treatment schedule used in the dose escalation study of F14512 [16]. The protocol consisted of three cycles of etoposide phosphate (ETOPOPHOS^®^) IV injections every 2 weeks. The total dose was divided in 3 administrations with a 3-hour injection once daily on 3 consecutive days (days 1–3, days 15–17, and days 29–31). Dogs were hospitalized 4 days during each cycle (days 1–4, days 15–18, and days 29–32).

The commercially available parenteral formulation of etoposide phosphate (ETOPOPHOS^®^) was used for IV administration. The prescribed dose was diluted with saline solution for a total volume of 50 ml and then immediately administered during a period of 3 hours. Etoposide phosphate was administered IV via an indwelling catheter inserted into a cephalic vein. No premedication was performed before the IV administration.

### Dog selection

Twenty-seven owned-dogs presenting with histologically or cytologically confirmed diagnosis of stage III-V high-grade lymphoma, were enrolled in the study. Dogs were considered eligible to received etoposide phosphate administration when they (i) had new or previously diagnosed high-grade lymphoma; (ii) had a measurable disease at the inclusion; (iii) had relapsed to standard therapies (including chemotherapy and/or glucocorticoids) or whose owners had declined standard therapies; (iv) had no anticancer treatment in the month before the inclusion (including steroids); (v) had no significant biochemical abnormality or cytopenia, which precluded the use of cytotoxic drugs; (vi) had no concurrent serious systemic disorder; (vii) had an expected survival time of at least 8 weeks, according to the veterinary subjective assessment.

Initial staging was performed at the time of the inclusion according to the World Health Organization (WHO) classification: five-stage criteria for canine lymphoma and lymph node size were assessed using published recommendations [29]. Staging tests included a complete blood count, chemistry panel, ionized calcium, two-view chest X-rays, an abdominal ultrasound, liver and spleen cytology, urine analysis and a bone marrow aspirate. Biopsy specimens from peripheral enlarged lymph nodes were fixed in 10% neutral-buffered formalin for 48 hours and embedded in paraffin wax. Four micrometer-thick sections were stained with hematoxylin and eosin. Immunophenotyping was performed on biopsies using antibodies targeting CD-3, used as a pan-T marker (monoclonal mouse anti-human F7.2.38; Dako) and an antibody targeting CD-20, used as a pan-B marker (rabbit anti-human RB-9013-P; Thermoscientific). Cells neoplasms negative for both CD20 and CD3 were also evaluated for PAX5 (clone 24; Cell Mark), and BLA36 (clone A27-42; Biogenex) expression (two antigens expressed by B-cell neoplasms).

This study protocol was approved by the OCR Ethical Committee. Written informed consent form was obtained from all clients. Owners were able to withdraw their dog from the study at any time.

### Assessment of toxicities

Safety and tolerability were assessed according to the Veterinary Cooperative Oncology Group criteria for adverse events (VCOG-CTCAE) [30]. According to the VCOG-CTCAE, the Dose Limiting Toxicity (DLT) was defined as: any grade 5 toxicity, any prolonged (>48 hours) asymptomatic grade 4 neutropenia, any grade 3 febrile neutropenia, and any grade 4 non-hematologic toxicity. Results of complete blood count, signs of gastrointestinal toxicosis or other constitutional clinical signs were recorded during and 1 week after each cycle. At each follow-up visit, owners were questioned about signs of adverse clinical effects, daily water intake, appetite, urination, vomiting, stool consistency/frequency, energy level, mood and exercise tolerance. Gastrointestinal adverse events were treated with symptomatic treatments. A prophylactic broad spectrum antibiotherapy was administrated in case of severe asymptomatic neutropenia (grades 3 and 4). Dogs with febrile neutropenia and severe gastrointestinal toxicity were hospitalized and treated with intravenous fluids and antibiotics. During drug administration, dogs were evaluated for hypersensitivity reactions. A physical examination was performed to identify any sign of agitation, head shaking, pruritis, acute cutaneous erythema, subcutaneous oedema. Heart rate, respiratory rate, body temperature were monitored and blood pressure was measured every hour during infusion.

### Assessment of response and follow-up

Complete remission (CR) was defined as complete regression of all measurable disease. Partial response (PR) was defined as >30% but <100% regression of measurable disease. Stable disease (SD) was defined as <30% reduction in size of all measurable disease or <20% increase in size of all measurable disease. Progressive disease (PD) was defined as an increase of >20% of measurable disease or the appearance of new lesions.

Peripheral lymph node size was measured every two weeks before each cycle according to published guidelines [31].

At day 45, dogs underwent complete end-staging (complete blood count, chemistry panel, two-view chest X-rays, and an abdominal ultrasound). Liver/spleen and bone marrow aspirates were performed in case of infiltration at the inclusion. No additional chemotherapy treatment was performed after the three cycles of etoposide phosphate and a follow up every 4 weeks was realized after the end-staging until relapse. The remission status was assessed by an oncologist on the basis of physical examination and peripheral lymph nodes size measurement. In case of relapse during the follow up, a complementary CHOP-based chemotherapy protocol was proposed to the dog's owner. The progression-free survival (PFS) was calculated from the date of treatment initiation to the date of PD. Due to the sample size of each cohorts, statistical analyses were not performed based of the PFS in this study. Kaplan–Meier estimation will be used to estimate and display the distribution of the PFS in the final phase II randomized double-blind clinical study of F14512 and etoposide phosphate in naturally occurring canine lymphoma.

## Results

### Epidemiologic characteristics and staging

Twenty-seven dogs with naturally occurring high-grade lymphoma were enrolled in this dose escalation trial between March and October 2014.

Epidemiologic characteristics are summarized in Table 2. Thirteen dogs were female; 14 were male. Median age was 7 years (range 3–14), and median body weight was 31 kg (range 4–66 kg). Eighteen different breeds were represented.

**Table 2:** Epidemiologic and clinical characteristics of the population studied (27 dogs).

Epidemiological characteristics			Population studied
Sex	Male		51.8% (14/27)
	Female		48.2% (13/27)
Age (years) (median [range])			7 [3–14]
Body weight (kg) (median [range])			31 [4–66]
Clinical characteristics	Breed	Labrador Retriever, Rottweiler, English Bulldog, Bullmastiff	7.4% (2/27 each 4 breeds)
		Shih-Tzu, Bernese Mountain Dog, Golden Retriever, Brittany, Berger de Bauce, French Bulldog, Dogue des Canaris, Boxer, Poodle, Weimaraner, Basset Artésien Normand, Swiss Shepherd, Yorkshire Terrier, English Setter	3.7% (1/27 each 14 breeds)
		Cross-breed	18.5% (5/27)
	WHO stage	Stage III	29.6% (8/27)
		Stage IV	63.0% (17/27)
		Stage V	7.4% (2/27)
	WHO substage	a	51.8% (14/27)
		b	48.2% (13/27)
	Lymphoma subtype	B cell	77.8% (21/27)
		T cell	18.5% (5/27)
		Unclassified	3.7% (1/27)
	Hypercalcemia		7.4% (2/27)
	Pretreatment	No treatment	48.1% (13/27)
		Corticosteroid	3.7% (1/27)
		Chemotherapy	48.1% (13/27)

Eight dogs (30%) were classified as clinical stage III, 17 (63%) as stage IV, and 2 (7%) as stage V. Fourteen dogs (52%) were substage a and 13 (48%) substage b. Twenty-one dogs (78%) were B-cell lymphoma and 5 (18%) were T-cell lymphoma. One dog was unclassified. Two dogs (7%) had hypercalcemia at the inclusion.

Thirteen dogs (48%) had no prior treatment, 13 (48%) dogs had been treated with chemotherapy and 1 dog received corticosteroids during 3 weeks before entering the study.

All dogs that had been treated with chemotherapy before entering the study had received only one prior chemotherapy protocol.

### Toxicities

All 27 dogs were evaluated for toxicities. Signs of toxicity included neutropenia, anemia, thrombocytopenia, and digestive disorders (diarrhea and vomiting). Toxicities are reported in Table 3.

**Table 3:** Toxicity observed in the population studied (27 dogs).

Cohort (dose)	Number of dogs	Hematologic Toxicity [Grade (number of dogs)]			Gastrointestinal Toxicity [Grade (number of dogs)]	
		Hemoglobin	Platelet	Neutrophils	Diarrhea	Vomiting
Cohort 1 (105 mg/m^2^)	3					
Cohort 2 (126 mg/m^2^)	5				Grade 1 (1)	
Cohort 3 (150 mg/m^2^)	4	Grade 1 (1)			Grade 2 (1)	Grade 2 (1)
Cohort 4 (180 mg/m^2^)	3	Grade 2 (2)	Grade 2 (1)		Grade 1 (2)	Grade 1 (2)
Cohort 5 (225 mg/m^2^)	4	Grade 3 (3)	Grade 1 (1)		Grade 1 (1)	Grade 1 (1)
Cohort 6 (300 mg/m^2^)	6	Grade 2 (1)	Grade 2 (2)	Grade 1 (1) Grade 2 (1) Grade 3 (1)	Grade 1 (2) Grade 2 (2)	Grade 1 (1) Grade 2 (3)
Cohort 7 (360 mg/m^2^)	2			Grade 4 (1)* **(DLT)**	Grade 4 (1) (**DLT)**	Grade 4 (1) (**DLT)**

*: Febrile neutropenia lasting more than 48 hours

DLT: Dose Limiting Toxicity

Twenty-three dogs (85%) had no neutropenic episode during the study. Four neutropenia toxicities were reported: one grade 1 in 1 dog (cohort 6), one grade 2 in 1 dog (cohort 6) one grade 3 in 1 dog (cohort 6) and one grade 4 in 1 dog (cohort 7). All grades 1 to 3 neutropenia were unique episodes for each dog, asymptomatic and reversible in 48 hours. The Grade 4 neutropenia was associated with pyrexia lasting more than 48 hours and was considered as a DLT (cohort 7). Neutropenia was observed 5 to 10 days (median: 8 days) after drug administration, Full hematologic recovery was confirmed at day 15 and day 29 before initiation of the next chemotherapy cycle.

Four dogs (15%) had a thrombocytopenic episode (1 Grade 1 and 3 Grade 2) and 7 dogs (26%) had haemoglobin toxicity (1 Grade 1, 3 Grade 2 and 3 Grade 3). Thrombocytopenia and haemoglobin toxicities were reported 8 to 14 days after drug administration. All hematologic toxicities were asymptomatic and reversible.

Gastrointestinal toxicities were reported in 13 dogs (48%). Five dogs had diarrhea, 2 dogs had vomiting and 6 dogs had vomiting and diarrhea. Ten gastrointestinal episodes were grade 1 (53%), 7 were grade 2 (37%), and 2 were grade 4 (10%) and occurred 2 to 8 days after drug administration. Grade 4 gastrointestinal toxicities were dose related and occurred in one dog from cohort 7 and were considered as a DLT. In this study no acute hypersensitivity reaction was observed.

Two (7%) dogs (all dogs from cohort 7) were hospitalized secondary to chemotherapy-induced toxicity, one dog because of grade 4 febrile neutropenia and one dog because of grade 4 gastrointestinal toxicities. The dog with grade 4 febrile neutropenia required a dose reduction (treated with the next lower dose level) and the dog with grade 4 digestive toxicities had a treatment discontinuation. No death related to treatment was reported.

### Clinical outcome

When considering all dogs irrespective of dose cohort, 2 (7.4%) dogs experienced a CR, 3 (11.1%) dogs experienced a PR and 7 (25.9%) dogs experienced a SD at D45 (Table 4). At the dose levels of 105, 126, 150, 180 and 225 mg/m^2^ a SD was observed in 6 dogs at D45 and 13 dogs had a PD before the end-staging. More than 3 dogs were enrolled in cohort n°2 (5 dogs), n°3 (4 dogs), and n°5 (4 dogs) where no DLT was observed because 2 dogs in cohort n°2, 1 dog in cohort n°3 and 1 dog in cohort n°5 experienced a PD 11 days, 15 days, 2 days and 5 days respectively, after the inclusion and therefore received only one cycle of etoposide phosphate. Additional dogs were enrolled in these cohorts to receive all cycles of etoposide phosphate administrations in order to determine the maximally tolerated dose. In the 6 dogs treated with dose level of 300 mg/m^2^, 2 dogs achieved a CR, 3 dogs a PR and one dog a SD at D45. At the dose level of 300 mg/m^2^, the overall response rate was 83.3% (5/6). Among the dogs treated with the dose level of 360 mg/m^2^ (n = 2), one dog had a PD at D45 and the other dog was excluded from the study on day 7 for severe toxicities (grade 4 gastrointestinal toxicosis).

**Table 4:** Clinical response of the population studied (27 dogs).

Population	Clinical response at day 45				PFS [Days (range)]
	PD	SD	PR	CR	
Wide population	51.8% (14/27)	25.9% (7/27)	11.1% (3/27)	7.4% (2/27)	55 (2–404)
Pretreatment					
Yes (n = 14) No (n = 13)	64.3% (9/14) 38.5% (5/13)	14.3% (2/14) 38.5% (5/13)	14.3% (2/14) 7.7% (1/13)	7.1% (1/14) 7.7% (1/13)	53 (2–270) 73 (5–404)
Cohort					
Cohort 1: 105 mg/m^2^ (n = 3) Cohort 2: 126 mg/m^2^ (n = 5) Cohort 3: 150 mg/m^2^ (n = 4) Cohort 4: 180 mg/m^2^ (n = 3) Cohort 5: 225 mg/m^2^ (n = 4) Cohort 6: 300 mg/m^2^ (n = 6) Cohort 7: 360 mg/m^2^ (n = 2)	33.3% (1/3) 80.0% (4/5) 75.0% (3/4) 33.3% (1/3) 100.0% (4/4) - 50.0% (1/2)	66.7% (2/3) 20.0% (1/5) 25.0% (1/4) 66.7% (2/3) - 16.7% (1/6) -	- - - - - 50.0% (3/6) -	- - - - - 33.3% (2/6) -	73 (16–245) 42 (11–90) 24.5 (2–240) 92 (45–101) 24 (5–53) 176 (60–404) 29

At the end of the protocol no additional chemotherapy treatment was performed and dogs were followed every 4 weeks until relapse. Eight dogs received rescue chemotherapy protocol after relapsing. No dog was alive at the time of data analysis. All dogs were euthanized or died secondary to their disease. The median time of follow up was 82 days (5–404 days) for all dogs, 49 days (5–404 days) for dogs who did not receive additional chemotherapy, and 92 days (20–282 days) for dogs with additional chemotherapy.

The median progression-free survival (PFS) for all dogs was 55 days (range 2–404 days). Dogs from cohort 6 (300 mg/m^2^) had the highest PFS (median: 176 days, range 60–404 days) (Table 4). There was no difference in PFS between: males versus females, age, B-cell lymphoma versus T-cell lymphoma, stage, substage a versus substage b and dogs that did or did not receive chemotherapy before the inclusion. The dog that had received corticosteroids was included in the prior chemotherapy group. Sixty-four percent (9/14) of dogs who had been treated with chemotherapy before entering the study had a relapse at D45 against 38% (5/13) of dogs with no prior treatment and all dogs who experienced a response (2 CR and 3 PR) had a B-cell lymphoma.

## Discussion

The objectives of this dose escalation study were to evaluate the safety and tolerability of intravenous etoposide phosphate (ETOPOPHOS^®^) injection in dogs with multicentric lymphoma. The maximal tolerated dose of etoposide phosphate in dogs was 300 mg/m^2^ when administered in 3 consecutive daily 3-hour infusions each of 100 mg/m^2^, every 2 weeks for three cycles. Only a moderate reversible gastrointestinal toxicity, no severe myelotoxicity and no hypersensitivity reaction were reported at this dose level. At this dose level an early sign of efficacy was assessed with an overall response rate of 83.3% (5/6) at D45 after three cycles of therapy. This study is, to the best of our knowledge, the first to report the safety and efficacy of etoposide phosphate in dogs with multicentric lymphoma.

Previous reports have investigated the efficacy and tolerability of etoposide administration in dogs with cancer [19,22,23]. One retrospective study on 13 dogs with relapsing lymphoma treated with single-agent etoposide showed a minimal therapeutic effect [23]. In this study, 13 dogs received 100 mg/m^2^ of etoposide as a single IV bolus or as daily IV bolus at 25 mg/m^2^ during four consecutive days every month. Only 2 of 13 (15%) dogs experienced a response (partial remission for eight days and three months respectively) and these 2 dogs received the 4-day schedule protocol. When dogs were treated with 100 mg/m^2^ as single IV bolus, none had a sustained response. The most severe adverse reaction after IV administration of etoposide was an acute pruritic cutaneous reaction that occurred in 11 of the 13 dogs (85%). Severe acute hypersensitivity reactions, caused by histamine release, have been reported in humans [32] and dogs treated with etoposide (VP-16) [23,33] or with drugs containing polysorbate-80 (docetaxel) [34,35]. Therefore, IV administration of etoposide in dogs was considered neither practical nor safe because of the adverse reactions observed. Oral administration of etoposide was studied with the parenteral formulation reconstituted with NaCl solution [22]. With a daily dose of 50 mg/m^2^ administered for 21 days, no adverse reactions were reported but oral bioavailability was low (median was 13.4%) and highly variable among dogs (range, 5.7% to 57.3%). Etoposide phosphate is a water-soluble pro-drug of etoposide formulated without polysorbate-80. Toxicokinetics and toxicodynamics of etoposide phosphate were investigated in beagle healthy dogs [21]. Doses 57 to 461 mg/m^2^ were administered following 5 min IV infusion. Pharmacokinetics evaluations showed that etoposide phosphate was rapidly and extensively converted to etoposide mediated by phosphatases present in serum and tissues. As in our study, myelosuppression was one of the major dose-limiting toxicities.

To determine the recommended dose of etoposide phosphate, we evaluated seven dose levels. The first cohort was treated at a starting dose of 105 mg/m^2^ with a daily IV bolus of 35 mg/m^2^ during three consecutive days. This first dose level was considered to be safe based on previous toxicological study [21]. All dogs from the cohort 1 to 5 readily tolerated the IV administration of etoposide phosphate. Due to the good tolerance at the dose levels of 105, 126, 150, 180 and 225 mg/m^2^ but a minimal observed therapeutic effect (n = 19, 13 PD and 6 SD at D45), it was decided to treat dogs in cohort 6 with a 30%-increase dose level (300 mg/m^2^). In this dose level, one asymptomatic and reversible (<24h) grade 3 neutropenia was reported in one dog and was not considered as a DLT. A prophylactic broad spectrum antibiotherapy was administrated to this dog and no hospitalization was recommended. In the dose escalation, asymptomatic grade 3 neutropenia was not considered as DLT according to the VCOG criteria [30]. No dogs had premedication before the IV administration of etoposide phosphate and no acute hypersensitivity reaction was observed during the study.

Severe gastrointestinal toxicities (grade 4) and severe myelotoxicities (grade 4 febrile neutropenia) were dose related and occurred in 2 dogs from cohort 7 and were considered as DLT. The dose escalation was discontinued and the recommended dose of 300 mg/m^2^ was defined as the dose level just below this toxic dose level. In addition, three more dogs were included and treated with the determined maximal tolerated dose to assess early signs of efficacy of this dose level (3+3 dogs in the cohort 6). The adverse effects observed in dogs are similar to the ones reported in human. Indeed, myelosuppression is dose related and dose limiting in human, with granulocyte and platelets nadirs occurring 7 to 14 days and 9 to 16 days after drug administration, respectively. Fever and infection have been reported in patients with neutropenia. Nausea and vomiting are the major gastrointestinal toxicities in human, with mild to moderate severity [36]. Both etoposide and its excipient (polysorbate-80) are suspected of causing hypersensitivity reactions in human [37]. Our study revealed that etoposide phosphate (that does not contain polysorbate-80) was better tolerated than etoposide in dogs, with a dose related and dose limiting toxicity.

At the dose levels of 105, 126, 150, 180 and 225 mg/m^2^ a minimal therapeutic effect was observed (n = 19, 6 SD at D45). However in the 6 dogs treated with the dose level of 300 mg/m^2^, 2 dogs achieved a CR and 3 dogs a PR at D45 with an overall response rate of 83.3% (5/6) and a progression-free survival of 176 days (range 60–404 days). Despite the limited number of dogs included in this study, these results suggest greater efficacy than the previous results reported on the use of etoposide in dogs with lymphoma (ORR: 15%, 2/13) [23]. However, a randomized phase II would be required to confirm this result. This discrepancy could be related to the decreased toxicity of etoposide phosphate compared to etoposide, which allowed us to use a higher and more efficacious dosage of the drug.

Etoposide cytotoxicity is mediated by inhibition of topoisomerase II. Antitumor activity of etoposide is phase-specific and has been reported to be schedule dependent. A superior activity has been demonstrated in human treated with etoposide when the total dose is given over consecutive days [38,39]. A randomized clinical trial evaluated the activity of 500 mg/m^2^ of single-agent etoposide IV administration as a 24-hour infusion or as a daily 2-hour infusion for 5 consecutive days in patients with small-cell lung cancer every 3 weeks [28]. Only 2 patients treated with 24-hour injection achieved a partial remission, resulting in an overall response rate of 10% (2/20). In the 5-day schedule, one patient had a CR and 16 a PR with an overall response rate of 89% (17/19), which was significantly superior. Pharmacokinetic data from this study revealed that duration of exposure to serum etoposide concentrations superior to 1 μg/mL was associated with a greater antitumor activity. Consequently in our dose escalation study, the response rate superior than previous studies could be explained by the dosing schedule of a daily 3-hour etoposide phosphate injection on 3 consecutive days, every 2 weeks. The treatment schedule used in this escalation study was the same as the one used in a previous pharmacology study of F14512 [16]. The number of daily administrations was lower than in the etoposide human study design. Indeed, it was decided to limit to 4 days the hospitalization time for dogs (with 3 daily infusions) and to repeat cycles every 2 weeks. A limit of this etoposide phosphate dose escalation study was the absence of assessment of pharmacokinetics and pharmacodynamics. Toxicokinetics and toxicodynamics were previously investigated in beagle healthy dogs receiving IV bolus of etoposide phosphate [21] but it would be interesting to evaluate the serum etoposide concentration in this 3-day schedule.

Multiagent chemotherapy is considered as the standard of care for treatment of non-Hodgkin’s-like lymphoma in dogs. CHOP-based protocol including cyclophosphamide, doxorubicin, vincristine and prednisolone, induces a remission of lymphoma in the majority of dogs, close to 90%, with a median remission from 6 to 12 month and survival times approximately of 12 months [40–44]. In single-agent chemotherapy, the remission and survival time are shorter than those achieved in CHOP-based protocols. Doxorubicin is commonly recognized as the most efficient single-agent for the treatment of canine lymphoma with response rates reported from 69% to 78% and median remission from 80.5 days to 206 days [45–50]. In a phase I dose escalation trial in canine lymphoma, we have previously showed that the response rate of F14512 was 91% [16]. In the study presented here, an overall response rate of 83% and a PFS of 176 days (range 60–404 days) were achieved in 6 dogs treated with 300 mg/m^2^. Therefore, these results are promising and showed the potential of etoposide phosphate, which could be further investigated either alone or in combination with other agents.

In conclusion, the maximally tolerated dose of etoposide phosphate (ETOPOPHOS^®^) in dogs was 300 mg/m^2^ with 3 consecutive daily 3-hour infusions each of 100 mg/m^2^, every 2 weeks. At this dose level, the overall response rate was 83.3% (5/6) at D45. Only a moderate reversible gastrointestinal toxicity, no severe myelotoxicity and no hypersensitivity reaction were reported at this dose level. Because the treatment schedule only included three cycles of etoposide phosphate injections, additional cycles could be considered in future studies to potentially increase clinical efficacy. This dose escalation study was a pilot translational research study to determine the recommended dose for a phase II randomized double-blind clinical study of F14512 and etoposide phosphate in naturally occurring canine lymphoma. This pilot study is, to the best of our knowledge, the first to report preliminary data on the safety and efficacy of etoposide phosphate in dogs with multicentric lymphoma. The results presented here will help diversify the therapeutic arsenal available for veterinarians, and, more importantly, will inform for future comparative oncology trials using the canine model of lymphoma to develop innovative therapies for humans. The ongoing phase II study will explore PK, PD and clinical efficacy with translational objectives for both dogs and humans.

## Supporting information

S1 File NC3Rs ARRIVE guidelines checklist. (PDF)
