# Supplementary material for: Dose escalation study to evaluate safety, tolerability and efficacy of intravenous etoposide phosphate administration in 27 dogs with multicentric lymphoma
Source: PLoS One. 2017 May 15;12(5):e0177486. doi: 10.1371/journal.pone.0177486 (PMC5432161; doi:10.1371/journal.pone.0177486)
Supplement: S1 File — (PDF) [file pone.0177486.s001.pdf]

The ARRIVE Guidelines Checklist

Animal Research: Reporting In Vivo Experiments

Carol Kilkenny[1], William J Browne[2], Innes C Cuthill[3], Michael Emerson[4] and Douglas G Altman[5]

[1]The National Centre for the Replacement, Refinement and Reduction of Animals in Research, London, UK, [2]School of Veterinary Science, University of Bristol, Bristol, UK, [3]School of Biological Sciences, University of Bristol, Bristol, UK, [4]National Heart and Lung Institute, Imperial College London, UK, [5]Centre for Statistics in Medicine, University of Oxford, Oxford, UK.

	ITEM	RECOMMENDATION	Section/ Paragraph
Title	1	Provide as accurate and concise a description of the content of the article as possible.	Dose escalation study to evaluate safety, tolerability and efficacy of intravenous etoposide phosphate administration in 27 dogs with multicentric lymphoma
Abstract	2	Provide an accurate summary of the background, research objectives, including details of the species or strain of animal used, key methods, principal findings and conclusions of the study.	BACKGROUND AND PURPOSE: Comparative oncology has shown that naturally occurring canine cancers are of valuable and translatable interest for the understanding of human cancer biology and the characterization of new therapies. This work was part of a comparative oncology project assessing a new, clinical-stage topoisomerase II inhibitor and comparing it with etoposide in dogs with spontaneous lymphoma with the objective to translate findings from dogs to humans. Etoposide is a topoisomerase II inhibitor widely used in various humans' solid and hematopoietic cancer, but little data is available concerning its potential antitumor efficacy in dogs. Etoposide phosphate is a water-soluble prodrug of etoposide which is expected to be better tolerated in dogs.<br>The objectives of this study were to assess the safety, the tolerability and the efficacy of intravenous etoposide phosphate in dogs with multicentric lymphoma.<br><br>EXPERIMENTAL APPROACH: Seven dose levels were evaluated in a traditional 3+3 phase I design. Twenty-seven owned-dogs with high-grade multicentric lymphoma were enrolled and treated with three cycles of etoposide phosphate IV injections every 2 weeks. Adverse effects were graded according to the Veterinary Cooperative Oncology Group criteria. A complete end-staging was realized 45 days after inclusion.<br><br>KEY RESULTS: The maximal tolerated dose was 300 mg/m^2. At this dose level, the overall response rate was 83.3% (n=6, 3 PR and 2 CR). Only a moderate reversible gastrointestinal toxicity, no severe myelotoxicity and no hypersensitivity reaction were reported at this dose level.<br><br>CONCLUSIONS AND IMPLICATIONS: Beyond the characterization of etoposide clinical efficacy in dogs, this study underlined the clinical and therapeutic homologies between dog and human lymphomas.

INTRODUCTION

Background	3	a. Include sufficient scientific background (including relevant references to previous work) to understand the motivation and context for the study, and explain the experimental approach and rationale.<br>b. Explain how and why the animal species and model being used can address the scientific objectives and, where appropriate, the study's relevance to human biology.	Etoposide, a semisynthetic derivative of podophyllotoxine, is a cytotoxic chemotherapy drug mediated by inhibition of topoisomerase II (3). Etoposide has been used for many years in humans for refractory or relapsed non-Hodgkin lymphoma in combination with other drugs (3–5).<br>This dose escalation study was determined as part of a comparative oncology project to compare the safety and the efficacy of F14512, a new polyamine-vectorized, podophyllotoxine-derivative anticancer drug, and etoposide phosphate in dogs with naturally occurring high-grade lymphoma. F14512 is a vectorized form of etoposide and has demonstrated *in vitro* and *in vivo* potent, superior to etoposide, antitumor activities in preclinical studies (6–13). Comparative oncology has revealed that spontaneous lymphomas in dogs share clinical, biologic, genetic, and therapeutic similarities with their human counterparts (14,15). A phase I clinical pharmacology study of F14512 in naturally occurring canine lymphoma was previously published to determine the recommended dose of F14512 in dogs (16). For comparative purposes, the safety, the tolerability and the efficacy of intravenous etoposide phosphate needed to be investigated in order to determine the recommended dose in dogs with multicentric lymphoma.<br><br>Etoposide is widely used in various humans' solid and hematopoietic cancers (3,17,18), but little data is available concerning its potential antitumor efficacy in dogs (19,20). Etoposide has been studied in dogs for pharmacokinetics and toxicologic purposes (21,22). Previous studies on canine lymphoma treated with intravenous administration of etoposide showed a minimal therapeutic effect associated with hematologic toxicity and severe acute hypersensitivity reactions probably associated with the vehicle (polysorbate-80) used for the parenteral formulation (23). This vehicle-related toxicosis was considered a limitation for the IV administration of etoposide in dogs. In humans, intravenous etoposide is generally well tolerated, but rare acute hypersensitivity reactions have been reported, manifested by dyspnea, chest discomfort, hypotension, bronchospasm and skin flushing (24,25). Patients who initially experienced a hypersensitivity reaction to intravenous etoposide tolerated the subsequent administration of intravenous etoposide phosphate without any allergic reaction (24,25).<br>Etoposide phosphate is a water soluble phosphate ester derivative of etoposide which does not contain polysorbate-80. This IV formulation is expected to be easier to use and better tolerated in dogs. *In vivo*, etoposide phosphate is rapidly and extensively converted to etoposide by phosphatases present in serum and tissues (21). In murine models, this pro-drug has shown a similar activity to etoposide in various tumors (26).
Objectives	4	Clearly describe the primary and any secondary objectives of the study, or specific hypotheses being tested.	Therefore, the objectives of this dose escalation clinical trial were (i) to assess the clinical and hematologic tolerance and determine the recommended dose of intravenous etoposide phosphate (ETOPOPHOS®) in dogs with naturally occurring high-grade lymphoma; and (ii) to identify early signs of efficacy of this treatment, with an evaluation of remission rate after three cycles of therapy.<br>This dose escalation study is a pilot translational research study for a future phase II randomized double-blind clinical study of F14512 and etoposide phosphate in naturally occurring canine lymphoma.
**METHODS**			
Ethical statement	5	Indicate the nature of the ethical review permissions, relevant licences (e.g. Animal [Scientific Procedures] Act 1986), and national or institutional guidelines for the care and use of animals, that cover the research.	This study protocol was approved by the Oncovet Clinical Research Ethical Committee. Written informed consent form was obtained from all owners.<br>Safety and tolerability were assessed according to the Veterinary Cooperative Oncology Group criteria for adverse events (VCOG-CTCAE) (30). Short-term response was assessed according to the RECIST criteria published for peripheral nodal lymphoma in dogs (31).

Study design	6	For each experiment, give brief details of the study design including:<br>a. The number of experimental and control groups.<br>b. Any steps taken to minimise the effects of subjective bias when allocating animals to treatment (e.g. randomisation procedure) and when assessing results (e.g. if done, describe who was blinded and when).<br>c. The experimental unit (e.g. a single animal, group or cage of animals).<br>A time-line diagram or flow chart can be useful to illustrate how complex study designs were carried out.	Twenty-seven owned-dogs presenting with histologically or cytologically confirmed diagnosis of stage III-V high-grade lymphoma, were enrolled in a traditional 3+3 phase I study. Dogs were considered eligible to received etoposide phosphate administration when they (i) had new or previously diagnosed high-grade lymphoma; (ii) had a measurable disease at the inclusion; (iii) had relapsed to standard therapies (including chemotherapy and/or glucocorticoids) or whose owners had declined standard therapies; (iv) had no anticancer treatment in the month before the inclusion (including steroids); (v) had no significant biochemical abnormality or cytopenia, which precluded the use of cytotoxic drugs; (vi) had no concurrent serious systemic disorder; (vii) had an expected survival time of at least 8 weeks, according to the veterinary subjective assessment.<br><br>Seven dose levels were evaluated in a traditional 3+3 phase I design: 105 mg/m^2 (cohort 1; n=3), 126 mg/m^2 (cohort 2; n=5), 150 mg/m^2 (cohort 3; n=4), 180 mg/m^2 (cohort 4; n=3), 225 mg/m^2 (cohort 5; n=4), 300 mg/m^2 (cohort 6; n=6) and 360 mg/m^2 (cohort 7; n=2).<br>In each cohort, a minimum of 3 dogs were included and received the same dose during the protocol. The first cohort was treated at a starting dose that was considered to be safe based on extrapolation from toxicological data (21) and the next cohorts were treated at increasing dose levels according to the following criteria: if no Dose Limiting Toxicity (DLT) was observed in the 3 dogs treated in a cohort, another three dogs could be treated at the next higher dose level. If one of the three dogs experienced a DLT, three more dogs were treated at the same dose level. The dose escalation was continued until at least two dogs among a cohort of three or six dogs experienced a DLT assessed according to the Veterinary Cooperative Oncology Group criteria for adverse events (VCOG-CTCAE) (30). The recommended dose was defined as the dose level just below this toxic dose level. In addition, after the last dog was included in the dose escalation study, three more dogs were included and treated with the maximal tolerated dose to assess early signs of efficacy at this dose level.<br><br>In the study, n refers to number of dogs, All dogs involved in the study followed the same protocol over a period of 6 weeks.<br><br>**Table 1: Dose escalation schedule.** The first cohort was treated at a starting dose of 105 mg/m^2 (daily dose of 35 mg/m^2) that was considered to be safe based on extrapolation from toxicological data in dogs (21). In the three following dose levels, a 20%- increase dose level was used. Due to the good tolerance at the dose levels n°1, 2, 3 and 4 but a minimal therapeutic effect observed, it was decided to treat dogs with a 25% and then a 33%-increase dose level in cohort n°5 and 6 respectively in order to reach the daily dose of 100 mg/m^2 reported in humans (28). In the cohort n°7, dogs were treated with the initial 20%-increase dose level.

Cohort No	Number of dogs	Single Dose	Total dose / cycle	% Dose escalation
1	3	35 mg/m^2	105 mg/m^2	Baseline
2	5	42 mg/m^2	126 mg/m^2	20%
3	4	50 mg/m^2	150 mg/m^2	19%
4	3	60 mg/m^2	180 mg/m^2	20%
5	4	75 mg/m^2	225 mg/m^2	25%
6	6	100 mg/m^2	300 mg/m^2	33%
7	2	120 mg/m^2	360 mg/m^2	20%

Experimental procedures

7

For each experiment and each experimental group, including controls, provide precise details of all procedures carried out. For example:

a. How (e.g. drug formulation and dose, site and route of administration, anaesthesia and analgesia used [including monitoring], surgical procedure, method of euthanasia). Provide details of any specialist equipment used, including supplier(s).

b. When (e.g. time of day).

c. Where (e.g. home cage, laboratory, water maze).

d. Why (e.g. rationale for choice of specific anaesthetic, route of administration, drug dose used).

All dogs included in the study had an initial staging performed at the time of the inclusion according to the World Health Organization (WHO) classification: five-stage criteria for canine lymphoma and lymph node size were assessed using published recommendations (29). Staging tests included a complete blood count, chemistry panel, ionized calcium, two-view chest X-rays, an abdominal ultrasound, liver and spleen cytology, urine analysis and a bone marrow aspirate and biopsies from peripheral enlarged lymph nodes. Initial staging was performed under general anesthesia (propofol 2-4 mg/kg, IV and isoflurane 1.5-2.5% mixed with 100% oxygen) and analgesia (morphine: 0.1-0.2 mg/kg, IV). Biopsy specimens from peripheral enlarged lymph nodes were fixed in 10% neutral-buffered formalin for 48 hours and embedded in paraffin wax. Four micrometer-thick sections were stained with hematoxylin and eosin. Immunophenotyping was performed on biopsies using antibodies targeting CD-3, used as a pan-T marker (monoclonal mouse anti-human F7.2.38; Dako) and an antibody targeting CD-20, used as a pan-B marker (rabbit anti-human RB-9013-P; Thermoscientific). Cells neoplasms negative for both CD20 and CD3 were also evaluated for PAX5 (clone 24; Cell Mark), and BLA36 (clone A27-42; Biogenex) expression (two antigens expressed by B-cell neoplasms).

The protocol consisted of three cycles of etoposide phosphate IV injections every 2 weeks. The total dose was divided in 3 administrations with a 3-hour injection once daily on 3 consecutive days (days 1–3, days 15–17, and days 29-31). The commercially available parenteral formulation of etoposide phosphate (ETOPOPHOS®) was used for IV administration. The prescribed dose was diluted with saline solution for a total volume of 50 ml and then immediately administered during a period of 3 hours. Etoposide phosphate was administered IV via an indwelling catheter inserted into a cephalic vein. No premedication was performed before the IV administration. During drug administration, dogs were evaluated for hypersensitivity reactions. A physical examination was performed to identify any sign of agitation, head shaking, pruritis, acute cutaneous erythema, subcutaneous oedema. Heart rate, respiratory rate, body temperature were monitored and blood pressure was measured every hour during infusion. Dogs were hospitalized 4 days during each cycle (days 1–4, days 15–18, and days 29-32).

At day 45, dogs underwent complete end-staging (complete blood count, chemistry panel, two-view chest X-rays, and an abdominal ultrasound). Liver/spleen and bone marrow aspirates were performed under general anesthesia (same protocol) in case of infiltration at the inclusion. No additional chemotherapy treatment was performed after the three cycles of etoposide phosphate and a follow up every 4 weeks was realized after the end-staging until relapse. The remission status was assessed by an oncologist on the basis of physical examination and peripheral lymph nodes size measurement. In case of relapse during the

			follow up, a complementary CHOP-based chemotherapy protocol was proposed to the dog's owner.<br><br>The intravenous (IV) route was used for etoposide phosphate injection based on extrapolation from toxicological data in dogs (21). Etoposide phosphate is a water soluble phosphate ester derivative of etoposide which does not contain polysorbate-80. This IV formulation is expected to be easier to use and better tolerated in dogs. *In vivo*, etoposide phosphate is rapidly and extensively converted to etoposide by phosphatases present in serum and tissues (21). In murine models, this pro-drug has shown a similar activity to etoposide in various tumors (26).
Experimental animals	8	a. Provide details of the animals used, including species, strain, sex, developmental stage (e.g. mean or median age plus age range) and weight (e.g. mean or median weight plus weight range).<br>b. Provide further relevant information such as the source of animals, international strain nomenclature, genetic modification status (e.g. knock-out or transgenic), genotype, health/immune status, drug or test naïve, previous procedures, etc.	Twenty-seven owned-dogs with naturally occurring high-grade lymphoma were enrolled in this dose escalation trial. Thirteen dogs were female; 14 were male. Median age was 7 years (range 3-14), and median body weight was 31 kg (range 4-66 kg). Eighteen different breeds were represented (Labrador Retriever, Rottweiler, English Bulldog, Bullmastiff , Shih-Tzu, Bernese Mountain Dog, Golden Retriever, Brittany, Berger de Bauce, French Bulldog, Dogue des Canaris, Boxer, Poodle, Weimaraner, Basset Artésien Normand, Swiss Shepherd, Yorkshire Terrier, English Setter).<br><br>Thirteen dogs (48%) had no prior treatment, 13 (48%) dogs had been treated with chemotherapy and 1 dog received corticosteroids during 3 weeks before entering the study.

Housing and husbandry	9	Provide details of:<br>a. Housing (type of facility e.g. specific pathogen free [SPF]; type of cage or housing; bedding material; number of cage companions; tank shape and material etc. for fish).<br>b. Husbandry conditions (e.g. breeding programme, light/dark cycle, temperature, quality of water etc for fish, type of food, access to food and water, environmental enrichment).<br>c. Welfare-related assessments and interventions that were carried out prior to, during, or after the experiment.	Dogs were hospitalized 4 days during each cycle (days 1–4, days 15–18, and days 29-32) in an individual oncology ward.<br><br>All dogs were allowed free access to water and a maintenance diet in a 12-hour light/dark cycle, and a temperature and humidity controlled ward.<br><br>Results of complete blood count, signs of gastrointestinal toxicosis or other constitutional clinical signs were recorded during and 1 week after each cycle. At each follow-up visit, owners were questioned about signs of adverse clinical effects, daily water intake, appetite, urination, vomiting, stool consistency/frequency, energy level, mood and exercise tolerance. Gastrointestinal adverse events were treated with symptomatic treatments. A prophylactic broad spectrum antibiotherapy was administrated in case of severe asymptomatic neutropenia (grades 3 and 4). Dogs with febrile neutropenia and severe gastrointestinal toxicity were hospitalized and treated with intravenous fluids and antibiotics. During drug administration, dogs were evaluated for hypersensitivity reactions. A physical examination was performed to identify any sign of agitation, head shaking, pruritis, acute cutaneous erythema, subcutaneous oedema. Heart rate, respiratory rate, body temperature were monitored and blood pressure was measured every hour during infusion.
Sample size	10	a. Specify the total number of animals used in each experiment, and the number of animals in each experimental group.<br>b. Explain how the number of animals was arrived at. Provide details of any sample size calculation used.<br>c. Indicate the number of independent replications of each experiment, if relevant.	Twenty-seven dogs with naturally occurring high-grade lymphoma were divided into seven dose levels with a traditional 3+3 phase I design: 105 mg/m^2 (cohort 1; n=3), 126 mg/m^2 (cohort 2; n=5), 150 mg/m^2 (cohort 3; n=4), 180 mg/m^2 (cohort 4; n=3), 225 mg/m^2 (cohort 5; n=4), 300 mg/m^2 (cohort 6; n=6) and 360 mg/m^2 (cohort 7; n=2).<br><br>A traditional 3+3 phase I design was used for the dose escalation study (27). In each cohort, a minimum of 3 dogs were included and received the same dose during the protocol. More than 3 dogs were enrolled in cohort n°2 (5 dogs), n°3 (4 dogs), and n°5 (4 dogs) where no DLT was observed because 2 dogs in cohort n°2, 1 dog in cohort n°3 and 1 dog in cohort n°5 experienced a PD 11 days, 15 days, 2 days and 5 days respectively, after the inclusion and therefore received only one cycle of etoposide phosphate. Additional dogs were enrolled in these cohorts to receive all cycles of etoposide phosphate administrations in order to determine the maximally tolerated dose. The dose escalation was continued until at least two dogs among a cohort of three or six dogs experienced a DLT assessed according to the Veterinary Cooperative Oncology Group criteria for adverse events (VCOG-CTCAE) (30). The recommended dose was defined as the dose level just below this toxic dose level. In addition, after the last dog was included in the dose escalation study, three more dogs were included and treated with the maximal tolerated dose to assess early signs of efficacy at this dose level.
Allocating animals to experimental groups	11	a. Give full details of how animals were allocated to experimental groups, including randomisation or matching if done.<br>b. Describe the order in which the animals in the different experimental groups were treated and assessed.	Dogs were considered eligible to received etoposide phosphate administration when they (i) had new or previously diagnosed high-grade lymphoma; (ii) had a measurable disease at the inclusion; (iii) had relapsed to standard therapies (including chemotherapy and/or glucocorticoids) or whose owners had declined standard therapies; (iv) had no anticancer treatment in the month before the inclusion (including steroids); (v) had no significant biochemical abnormality or cytopenia, which precluded the use of cytotoxic drugs; (vi) had no concurrent serious systemic disorder; (vii) had an expected survival time of at least 8 weeks, according to the veterinary subjective assessment.<br>In each cohort, a minimum of 3 dogs were included and received the same dose during the protocol. The first cohort was treated at a starting dose that was considered to be safe based on extrapolation from

			toxicological data (21) and the next cohorts were treated at increasing dose levels according to the following criteria: if no Dose Limiting Toxicity (DLT) was observed in the 3 dogs treated in a cohort, another three dogs could be treated at the next higher dose level. If one of the three dogs experienced a DLT, three more dogs were treated at the same dose level. The dose escalation was continued until at least two dogs among a cohort of three or six dogs experienced a DLT assessed according to the Veterinary Cooperative Oncology Group criteria for adverse events (VCOG-CTCAE) (30). The recommended dose was defined as the dose level just below this toxic dose level.
Experimental outcomes	12	Clearly define the primary and secondary experimental outcomes assessed (e.g. cell death, molecular markers, behavioural changes).	Two primary outcome measures were assessed: clinical and hematologic tolerance and the maximally tolerated dose of intravenous etoposide phosphate (ETOPOPHOS®) in dogs with naturally occurring high-grade lymphoma;<br>In addition, one secondary outcome measure was evaluated: the efficacy of this treatment, with an evaluation of remission rate after three cycles of therapy (day 45).
Statistical methods	13	a. Provide details of the statistical methods used for each analysis.<br>b. Specify the unit of analysis for each dataset (e.g. single animal, group of animals, single neuron).<br>c. Describe any methods used to assess whether the data met the assumptions of the statistical approach.	Continuous data were expressed as means (range) and percentages. The progression-free survival (PFS) was calculated from the date of treatment initiation to the date of PD. Due to the sample size of each cohorts, statistical analyses were not performed based of the PFS in this study. Kaplan–Meier estimation will be used to estimate and display the distribution of the PFS in the final phase II randomized double-blind clinical study of F14512 and etoposide phosphate in naturally occurring canine lymphoma.
RESULTS			
Baseline data	14	For each experimental group, report relevant characteristics and health status of animals (e.g. weight, microbiological status, and drug or test naïve) prior to treatment or testing. (This information can often be tabulated).	Twenty-seven dogs with naturally occurring high-grade lymphoma were enrolled in this dose escalation trial. Thirteen dogs were female; 14 were male. Median age was 7 years (range 3-14), and median body weight was 31 kg (range 4-66 kg). Eighteen different breeds were represented. All dogs included in the study had an initial staging performed at the time of the inclusion according to the World Health Organization (WHO) classification: five-stage criteria for canine lymphoma and lymph node size were assessed using published recommendations (29). Eight dogs (30%) were classified as clinical stage III, 17 (63%) as stage IV, and 2 (7%) as stage V. Fourteen dogs (52%) were substage a and 13 (48%) substage b. Twenty-one dogs (78%) were B-cell lymphoma and 5 (18%) were T-cell lymphoma. One dog was unclassified. Two dogs (7%) had hypercalcemia at the inclusion. Thirteen dogs (48%) had no prior treatment, 13 (48%) dogs had been treated with chemotherapy and 1 dog received corticosteroids during 3 weeks before entering the study.
Numbers analysed	15	a. Report the number of animals in each group included in each analysis. Report absolute numbers (e.g. 10/20, not 50%[2]).<br>b. If any animals or data were not included in the analysis, explain why.	Three dogs were include in the cohort n°1 (3/27), 5 dogs in the cohort n°2 (5/27), 4 dogs in the cohort n°3 (4/27), 3 dogs in the cohort n°4 (3/27), 4 dogs in the cohort n°5 (4), 6 dogs in the cohort n°6 (6/27) and 2 dogs in the cohort n°7 (2/27) (Table 1).
Outcomes and estimation	16	Report the results for each analysis carried out, with a measure of precision (e.g. standard error or confidence interval).	When considering all dogs irrespective of dose cohort, 2 (2/27) dogs experienced a CR, 3 (3/27) dogs experienced a PR and 7 (7/27) dogs experienced a SD at D45 (Table 2). At the dose levels of 105, 126, 150, 180 and 225 mg/m^2 a SD was observed in 6 dogs at D45 and 13 dogs had a PD before the end-staging. In the 6 dogs treated with dose level of 300 mg/m^2, 2 dogs achieved a CR, 3 dogs a PR and one dog a SD at D45. At the dose level of 300 mg/m^2, the overall response rate was 83.3% (5/6). Among the dogs treated with the dose level of 360 mg/m^2 (n=2), one dog had a PD at D45 and the other dog was excluded from the study on day 7 for severe toxicities (grade 4 gastrointestinal toxicosis).<br>At the end of the protocol no additional chemotherapy treatment was performed and dogs were followed

every 4 weeks until relapse. Eight dogs received rescue chemotherapy protocol after relapsing. No dog was alive at the time of data analysis. All dogs were euthanized or died secondary to their disease. The median time of follow up was 82 days (5-404 days) for all dogs, 49 days (5-404 days) for dogs who did not receive additional chemotherapy, and 92 days (20-282 days) for dogs with additional chemotherapy.

The median progression-free survival (PFS) for all dogs was 55 days (range 2-404 days). Dogs from cohort 6 (300 mg/m^2) had the highest PFS (median: 176 days, range 60-404 days) (Table 2). There was no difference in PFS between: males versus females, age, B-cell lymphoma versus T-cell lymphoma, stage, substage a versus substage b and dogs that did or did not receive chemotherapy before the inclusion. The dog that had received corticosteroids was included in the prior chemotherapy group. Sixty-four percent (9/14) of dogs who had been treated with chemotherapy before entering the study had a relapse at D45 against 38% (5/13) of dogs with no prior treatment and all dogs who experienced a response (2 CR and 3 PR) had a B-cell lymphoma.

Table 2: Clinical response of the population studied (27 dogs)

Population	Clinical response at day 45				PFS
	PD	SD	PR	CR	Days (range)
Wide population	51.8% (14/27)	25.9% (7/27)	11.1% (3/27)	7.4% (2/27)	55 (2-404)
Pretreatment					
Yes (n=14)	64.3% (9/14)	14.3% (2/14)	14.3% (2/14)	7.1% (1/14)	53 (2-270)
No (n=13)	38.5% (5/13)	38.5% (5/13)	7.7% (1/13)	7.7% (1/13)	73 (5-404)
Cohort					
Cohort 1: 105 mg/m^2 (n=3)	33.3% (1/3)	66.7% (2/3)	-	-	73 (16-245)
Cohort 2: 126 mg/m^2 (n=5)	80.0% (4/5)	20.0% (1/5)	-	-	42 (11-90)
Cohort 3: 150 mg/m^2 (n=4)	75.0% (3/4)	25.0% (1/4)	-	-	24.5 (2-240)
Cohort 4: 180 mg/m^2 (n=3)	33.3% (1/3)	66.7% (2/3)	-	-	92 (45-101)
Cohort 5: 225 mg/m^2 (n=4)	100.0% (4/4)	-	-	-	24 (5-53)
Cohort 6: 300 mg/m^2 (n=6)	-	16.7% (1/6)	50.0% (3/6)	33.3% (2/6)	176 (60-404)
Cohort 7: 360 mg/m^2 (n=2)	50.0% (1/2)	-	-	-	29

Adverse events | 17 | a. Give details of all important adverse events in each experimental group.
b. Describe any modifications to the experimental protocols made to reduce adverse events.

All 27 dogs were evaluated for toxicities. Safety and tolerability were assessed according to the Veterinary Cooperative Oncology Group criteria for adverse events (VCOG-CTCAE) (30). Signs of toxicity included neutropenia, anemia, thrombocytopenia, and digestive disorders (diarrhea and vomiting).

Twenty-three dogs (85%) had no neutropenic episode during the study. Four neutropenia toxicities were reported: one grade 1 in 1 dog (cohort 6), one grade 2 in 1 dog (cohort 6) one grade 3 in 1 dog (cohort 6) and one grade 4 in 1 dog (cohort 7). All grades 1 to 3 neutropenia were unique episodes for each dog,

asymptomatic and reversible in 48 hours. The Grade 4 neutropenia was associated with pyrexia lasting more than 48 hours and was considered as a DLT (cohort 7). Neutropenia was observed 5 to 10 days (median: 8 days) after drug administration, Full hematologic recovery was confirmed at day 15 and day 29 before initiation of the next chemotherapy cycle.
Four dogs (15%) had a thrombocytopenic episode (1 Grade 1 and 3 Grade 2) and 7 dogs (26%) had haemoglobin toxicity (1 Grade 1, 3 Grade 2 and 3 Grade 3). Thrombocytopenia and haemoglobin toxicities were reported 8 to 14 days after drug administration. All hematologic toxicities were asymptomatic and reversible.
Gastrointestinal toxicities were reported in 13 dogs (48%). Five dogs had diarrhea, 2 dogs had vomiting and 6 dogs had vomiting and diarrhea. Ten gastrointestinal episodes were grade 1 (53%), 7 were grade 2 (37%), and 2 were grade 4 (10%) and occurred 2 to 8 days after drug administration. Grade 4 gastrointestinal toxicities were dose related and occurred in one dog from cohort 7 and were considered as a DLT. In this study no acute hypersensitivity reaction was observed.

Two (7%) dogs (all dogs from cohort 7) were hospitalized secondary to chemotherapy-induced toxicity, one dog because of grade 4 febrile neutropenia and one dog because of grade 4 gastrointestinal toxicities. The dog with grade 4 febrile neutropenia required a dose reduction (treated with the next lower dose level) and the dog with grade 4 digestive toxicities had a treatment discontinuation. No death related to treatment was reported.

DISCUSSION

Item	No.	Recommendation	
Interpretation/ scientific implications	18	a. Interpret the results, taking into account the study objectives and hypotheses, current theory and other relevant studies in the literature.<br>b. Comment on the study limitations including any potential sources of bias, any limitations of the animal model, and the imprecision associated with the results[2].<br>c. Describe any implications of your experimental methods or findings for the replacement, refinement or reduction (the 3Rs) of the use of animals in research.	The objectives of this dose escalation study were to evaluate the safety and tolerability of intravenous etoposide phosphate (ETOPOPHOS®) injection in dogs with multicentric lymphoma. The maximal tolerated dose of etoposide phosphate in dogs was 300 mg/m^2 when administered in 3 consecutive daily 3-hour infusions each of 100 mg/m^2, every 2 weeks for three cycles. Only a moderate reversible gastrointestinal toxicity, no severe myelotoxicity and no hypersensitivity reaction were reported at this dose level. At this dose level an early sign of efficacy was assessed with an overall response rate of 83.3% (5/6) at D45 after three cycles of therapy. This study is, to the best of our knowledge, the first to report the safety and efficacy of etoposide phosphate in dogs with multicentric lymphoma.<br><br>Previous reports have investigated the efficacy and tolerability of etoposide administration in dogs with cancer (19,22,23). One retrospective study on 13 dogs with relapsing lymphoma treated with single-agent etoposide showed a minimal therapeutic effect (23). In this study, 13 dogs received 100 mg/m^2 of etoposide as a single IV bolus or as daily IV bolus at 25 mg/m^2 during four consecutive days every month. Only 2 of 13 (15%) dogs experienced a response (partial remission for eight days and three months respectively) and these 2 dogs received the 4-day schedule protocol. When dogs were treated with 100 mg/m^2 as single IV bolus, none had a sustained response. The most severe adverse reaction after IV administration of etoposide was an acute pruritic cutaneous reaction that occurred in 11 of the 13 dogs (85%). Severe acute hypersensitivity reactions, caused by histamine release, have been reported in humans (31) and dogs treated with etoposide (VP-16) (23,33) or with drugs containing polysorbate-80 (docetaxel) (34,35). Therefore, IV administration of etoposide in dogs was considered neither practical nor safe because of the adverse reactions observed. Oral administration of etoposide was studied with the parenteral formulation reconstituted with NaCl solution (22). With a daily dose of 50 mg/m^2 administered for 21 days, no adverse reactions were reported but oral bioavailability was low (median was 13.4%) and highly variable among dogs (range, 5.7% to 57.3%). Etoposide phosphate is a water-soluble pro-drug of etoposide formulated

			without polysorbate-80. Toxicokinetics and toxicodynamics of etoposide phosphate were investigated in beagle healthy dogs (21). Doses 57 to 461 mg/m^2 were administered following 5 min IV infusion. Pharmacokinetics evaluations showed that etoposide phosphate was rapidly and extensively converted to etoposide mediated by phosphatases present in serum and tissues. As in our study, myelosuppression was one of the major dose-limiting toxicities.<br>Despite the limited number of dogs included in this study, these results suggest greater efficacy than the previous results reported on the use of etoposide in dogs with lymphoma (ORR: 15%, 2/13) (23). However, a randomized phase II would be required to confirm this result. This discrepancy could be related to the decreased toxicity of etoposide phosphate compared to etoposide, which allowed us to use a higher and more efficacious dosage of the drug.<br><br>A limit of this etoposide phosphate dose escalation study was the absence of assessment of pharmacokinetics and pharmacodynamics. Toxicokinetics and toxicodynamics were previously investigated in beagle healthy dogs receiving IV bolus of etoposide phosphate (21) but it would be interesting to evaluate the serum etoposide concentration in this 3-day schedule.
Generalisability/ translation	19	Comment on whether, and how, the findings of this study are likely to translate to other species or systems, including any relevance to human biology.	Comparative oncology has shown that naturally occurring canine cancers are of valuable and translatable interest for the understanding of human cancer biology and the characterization of new therapies. This work was part of a comparative oncology project assessing a new, clinical-stage topoisomerase II inhibitor and comparing it with etoposide in dogs with spontaneous lymphoma with the objective to translate findings from dogs to humans. Etoposide is a topoisomerase II inhibitor widely used in various humans' solid and hematopoietic cancer, but little data is available concerning its potential antitumor efficacy in dogs. Etoposide phosphate is a water-soluble prodrug of etoposide which is expected to be better tolerated in dogs. Beyond the characterization of etoposide clinical efficacy in dogs, this study underlined the clinical and therapeutic homologies between dog and human lymphomas.
Funding	20	List all funding sources (including grant number) and the role of the funder(s) in the study.	This work was supported by a grant from BPI-France, through the CaninCa project and the collaboration with the Pierre Fabre Research Institute.

NC3Rs

References:

1. Siegel R, Naishadham D, Jemal A. Cancer statistics, 2013. *CA Cancer J Clin* 2013; 63(1): 11-30.

2. Younes A, Thieblemont C, Morschhauser F, Flinn I, Friedberg JW, Amorim S, et al. Combination of ibrutinib with rituximab, cyclophosphamide, doxorubicin, vincristine, and prednisone (R-CHOP) for treatment-naive patients with CD20-positive B-cell non-Hodgkin lymphoma: a non-randomised, phase 1b study. *Lancet Oncol* 2014; 15(9): 1019-26.

3. Hande KR. Etoposide: four decades of development of a topoisomerase II inhibitor. *Eur J Cancer* 1998; 34(10): 1514-21.

4. Doorduijn JK, Spruit P, van Der Holt B, van't Veer M, Budel L, Löwenberg B, et al. Etoposide, mitoxantrone and prednisone: a salvage regimen with low toxicity for refractory or relapsed non-Hodgkin's lymphoma. *Haematologica* 2000; 85(8): 814-9.

5. Bo L-J, Liang A-B, Liu B, Chen Y-H, Wang F, Jin X-P. Efficacy of DICE (dexamethasone, etoposide, ifosfamide, and cisplatin) regimen on recurrent and refractory non-Hodgkin's lymphoma. *Chin J Cancer* 2006; 25(12): 1553-6.

6. Barret J-M, Kruczynski A, Vispé S, Annereau J-P, Brel V, Guminski Y, et al. F14512, a potent antitumor agent targeting topoisomerase II vectored into cancer cells via the polyamine transport system. *Cancer Res* 2008; 68(23): 9845-53.

7. Annereau J-P, Brel V, Dumontet C, Guminski Y, Imbert T, Broussas M, et al. A fluorescent biomarker of the polyamine transport system to select patients with AML for F14512 treatment. *Leuk Res* 2010; 34(10): 1383-9.

8. Brel V, Annereau J-P, Vispé S, Kruczynski A, Bailly C, Guilbaud N. Cytotoxicity and cell death mechanisms induced by the polyamine-vectorized anti-cancer drug F14512 targeting topoisomerase II. *Biochem Pharmacol* 2011; 82(12): 1843-52.

9. Gentry AC, Pitts SL, Jablonsky MJ, Bailly C, Graves DE, Osheroff N. Interactions between the etoposide derivative F14512 and human type II topoisomerases: implications for the C4 spermine moiety in promoting enzyme-mediated DNA cleavage. *Biochemistry* 2011; 50(15): 3240-9.

10. Kruczynski A, Vandenberghe I, Pillon A, Pesnel S, Goetsch L, Barret J-M, et al. Preclinical activity of F14512, designed to target tumors expressing an active polyamine transport system. *Invest New Drugs* 2011; 29(1): 9-21.

11. Kruczynski A, Pillon A, Créancier L, Vandenberghe I, Gomes B, Brel V, et al. F14512, a polyamine-vectorized anti-cancer drug, currently in clinical trials exhibits a marked preclinical anti-leukemic activity. *Leukemia* 2013; 27(11): 2139-48.

12. Mouawad F, Gros A, Rysman B, Bal-Mahieu C, Bertheau C, Horn S, et al. The antitumor drug F14512 enhances cisplatin and ionizing radiation effects in head and neck squamous carcinoma cell lines. *Oral Oncol* 2014; 50(2): 113-9.

13. Leblond P, Boulet E, Bal-Mahieu C, Pillon A, Kruczynski A, Guilbaud N, et al. Activity of the polyamine-vectorized anti-cancer drug F14512 against pediatric glioma and neuroblastoma cell lines. *Invest New Drugs* 2014; 32(5): 883-92.

14. Ponce F, Marchal T, Magnol JP, Turinelli V, Ledieu D, Bonnefont C, et al. A morphological study of 608 cases of canine malignant lymphoma in France with a focus on comparative similarities between canine and human lymphoma morphology. *Vet Pathol* 2010; 47(3): 414-33.

15. Ranieri G, Gadaleta CD, Patruno R, Zizzo N, Daidone MG, Hansson MG, et al. A model of study for human cancer: Spontaneous occurring tumors in dogs. Biological features and translation for new anticancer therapies. *Crit Rev Oncol Hematol* 2013; 88(1): 187-97.

16. Tierny D, Serres F, Segaoula Z, Bemelmans I, Bouchaert E, Pétain A, et al. Phase I Clinical Pharmacology Study of F14512, a New Polyamine-Vectorized Anticancer Drug, in Naturally Occurring Canine Lymphoma. *Clin Cancer Res* 2015; 21(23): 5314-23.

17. Montecucco A, Biamonti G. Cellular response to etoposide treatment. *Cancer Lett* 2007; 252(1): 9-18.

18. Joel S. The clinical pharmacology of etoposide: an update. *Cancer Treat Rev* 1996; 22(3): 179-221.

19. Lana S, U'ren L, Plaza S, Elmslie R, Gustafson D, Morley P, et al. Continuous low-dose oral chemotherapy for adjuvant therapy of splenic hemangiosarcoma in dogs. *J Vet Intern Med* 2007; 21(4): 764-9.

20. Willmann M, Müllauer L, Schwendenwein I, Wolfesberger B, Kleiter M, Pagitz M, et al. Chemotherapy in canine acute megakaryoblastic leukemia: a case report and review of the literature. *Vivo* 2009; 23(6): 911-8.

21. Igwemezie LN, Kaul S, Barbhaiya RH. Assessment of toxicokinetics and toxicodynamics following intravenous administration of etoposide phosphate in beagle dogs. *Pharm Res* 1995; 12(1): 117-23.

22. Flory AB, Rassnick KM, Balkman CE, Kiselow MA, Autio K, Beaulieu BB, et al. Oral bioavailability of etoposide after administration of a single dose to tumor-bearing dogs. *Am J Vet Res* 2008; 69(10): 1316-22.

23. Hohenhaus AE, Matus RE. Etoposide (VP-16). Retrospective analysis of treatment in 13 dogs with lymphoma. *J Vet Intern Med* 1990; 4(5): 239-41.

24. Siderov J, Prasad P, De Boer R, Desai J. Safe administration of etoposide phosphate after hypersensitivity reaction to intravenous etoposide. *Br J Cancer* 2002; 86(1): 12-3.

25. Collier K, Schink C, Young AM, How K, Seckl M, Savage P. Successful treatment with etoposide phosphate in patients with previous etoposide hypersensitivity. *J Oncol Pharm Pract* 2008; 14(1): 51‐5.

26. Rose WC, Basler GA, Trail PA, Saulnier M, Crosswell AR, Casazza AM. Preclinical antitumor activity of a soluble etoposide analog, BMY-40481-30. *Invest New Drugs* 1990;8 Suppl 1:S25-32.

27. Le Tourneau C, Lee JJ, Siu LL. Dose escalation methods in phase I cancer clinical trials. *J Natl Cancer Inst* 2009; 101(10): 708‐20.

28. Slevin ML, Clark PI, Joel SP, Malik S, Osborne RJ, Gregory WM, et al. A randomized trial to evaluate the effect of schedule on the activity of etoposide in small-cell lung cancer. *J Clin Oncol* 1989; 7(9): 1333‐40.

29. Valli VE, San Myint M, Barthel A, Bienzle D, Caswell J, Colbatzky F, et al. Classification of canine malignant lymphomas according to the World Health Organization criteria. *Vet Pathol* 2011; 48(1): 198‐211.

30. Veterinary cooperative oncology group - common terminology criteria for adverse events (VCOG-CTCAE) following chemotherapy or biological antineoplastic therapy in dogs and cats v1.1 [published online ahead of print July, 2011]. *Vet Comp Oncol.* doi: 10.1111/vco.283.

31. Vail DM, Michels GM, Khanna C, Selting KA, London CA, Veterinary Cooperative Oncology Group. Response evaluation criteria for peripheral nodal lymphoma in dogs (v1.0)--a Veterinary Cooperative Oncology Group (VCOG) consensus document. *Vet Comp Oncol* 2010; 8(1): 28‐37.

32. Masini E, Planchenault J, Pezziardi F, Gautier P, Gagnol JP. Histamine-releasing properties of Polysorbate 80 in vitro and in vivo: correlation with its hypotensive action in the dog. *Agents Actions* 1985; 16(6): 470‐7.

33. Ogilvie GK, Cockburn CA, Tranquilli WJ, Reschke RW, Weigel RM. Hypotension and cutaneous reactions associated with intravenous administration of etoposide in the dog. *Am J Vet Res* 1988; 49(8): 1367‐70.

34. Gough WB, Zeiler RH, Barreca P, El-Sherif N. Hypotensive action of commercial intravenous amiodarone and polysorbate 80 in dogs. *J Cardiovasc Pharmacol* 1982; 4(3): 375‐80.

35. McEntee M, Silverman JA, Rassnick K, Zgola M, Chan AO, Tau PT, et al. Enhanced bioavailability of oral docetaxel by co-administration of cyclosporin A in dogs and rats. *Vet Comp Oncol* 2003; 1(2): 105‐12.
